# Primary Cutaneous Epstein-Barr Virus (EBV)-Positive Diffuse Large B-cell Lymphoma With Synchronous Systemic T-cell Lymphoma: A Case Report

**DOI:** 10.7759/cureus.87441

**Published:** 2025-07-07

**Authors:** João Soares, João Teixeira, Carolina Afonso, Joana Calvão, Maria Manuel Xavier Brites, Jose C Cardoso

**Affiliations:** 1 Dermatology, University Hospital, Unidade Local de Saúde de Coimbra, Coimbra, PRT; 2 Hematology, University Hospital, Unidade Local de Saúde de Coimbra, Coimbra, PRT

**Keywords:** case report, diffuse large b-cell lymphoma, ebv, r-chop, t-cell lymphoma

## Abstract

Epstein-Barr virus (EBV)-positive diffuse large B-cell lymphoma (DLBCL) is a rare and aggressive lymphoma that has been associated with age-related immunosenescence. Here, we present the case of a 79-year-old man with violaceous nodules on the skin, mainly on the lower and upper limbs, diagnosed as primary cutaneous EBV-positive diffuse large B-cell lymphoma (DLBCL) based on histopathological and immunohistochemical findings. Systemic evaluation revealed synchronous peripheral T-cell lymphoma, not otherwise specified (NOS), without systemic B-cell lymphoma involvement. The patient was treated with rituximab, cyclophosphamide, hydroxydaunorubicin, vincristine (Oncovin), and prednisone (R-CHOP) chemotherapy, achieving a complete response for both lymphomas, with sustained outcomes after one year of follow-up. This case highlights the importance of age as a risk factor for EBV-related malignancies, the role of Epstein-Barr encoding region (EBER) and cluster of differentiation 30 (CD30) testing in diagnosis, and the potential effectiveness of R-CHOP in treating this rare lymphoma association. Further research is needed to establish optimal management strategies.

## Introduction

Epstein-Barr virus (EBV) infects more than 90% of the global population [1]. While it is commonly associated with infectious mononucleosis, most infections occur asymptomatically during childhood through salivary contact [2]. The virus primarily replicates in oral epithelium and later demonstrates a tropism for B cells, contributing to malignancies in adulthood. EBV is implicated in approximately 200,000 new cancer cases annually, predominantly involving B-cell malignancies [3].

EBV-associated lymphomas are generally more aggressive [4,5]. Their occurrence has been strongly linked to immunodeficiency states, such as primary immunodeficiencies, posttransplant immunosuppression, and HIV/AIDS. However, cases of EBV-related B-cell lymphoproliferative disorders have also been reported in the absence of overt immunodeficiency. In such cases, age-related immunosenescence is proposed as a significant contributing factor [5-7]. Lymphomas associated with EBV in this context tend to exhibit heightened aggressiveness [8].

EBV-positive diffuse large B-cell lymphoma (DLBCL) is a large B-cell lymphoma in which the majority of the neoplastic cells harbor EBV [9-11]. It is a rare entity, with limited data available regarding its diagnosis, progression, and treatment [7,12,13]. Even more infrequent is its association with synchronous systemic T-cell lymphoma, a combination that has seldom been described in the literature before.

We report the case of a 79-year-old immunocompetent man diagnosed with a primary cutaneous EBV-positive diffuse large B-cell lymphoma, with concurrent systemic peripheral T-cell lymphoma, not otherwise specified (NOS), an association that is exceedingly rare. Although a few cases of primary cutaneous EBV-positive DLBCL have been described, the coexistence with a synchronous systemic T-cell lymphoma has not been systematically studied and is scarcely reported in the literature [14]. This report contributes to the limited understanding of such dual lymphomas, highlights the role of EBV and cluster of differentiation 30 (CD30) testing in cutaneous lymphoproliferative disorders, and documents a favorable response to rituximab, cyclophosphamide, hydroxydaunorubicin, vincristine (Oncovin), and prednisone (R-CHOP) chemotherapy, which may inform future management strategies.

## Case presentation

**Figure 1:**
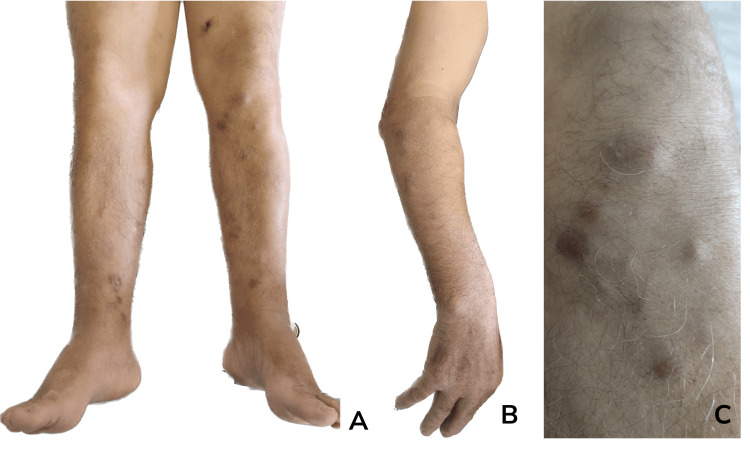
Violaceous nodules on the lower limbs (A), on the right forearm (B), and on the left leg (C) of the patient.

A 79-year-old man presented with non-painful violaceous nodules on the lower and upper limbs that had progressively increased in number and size over the preceding eight months (Figure 1). The patient did not report fever, night sweats, or weight loss. His medical history was unremarkable, with no prior HIV infection, organ transplantation, or use of immunosuppressive medications.

**Figure 2:**
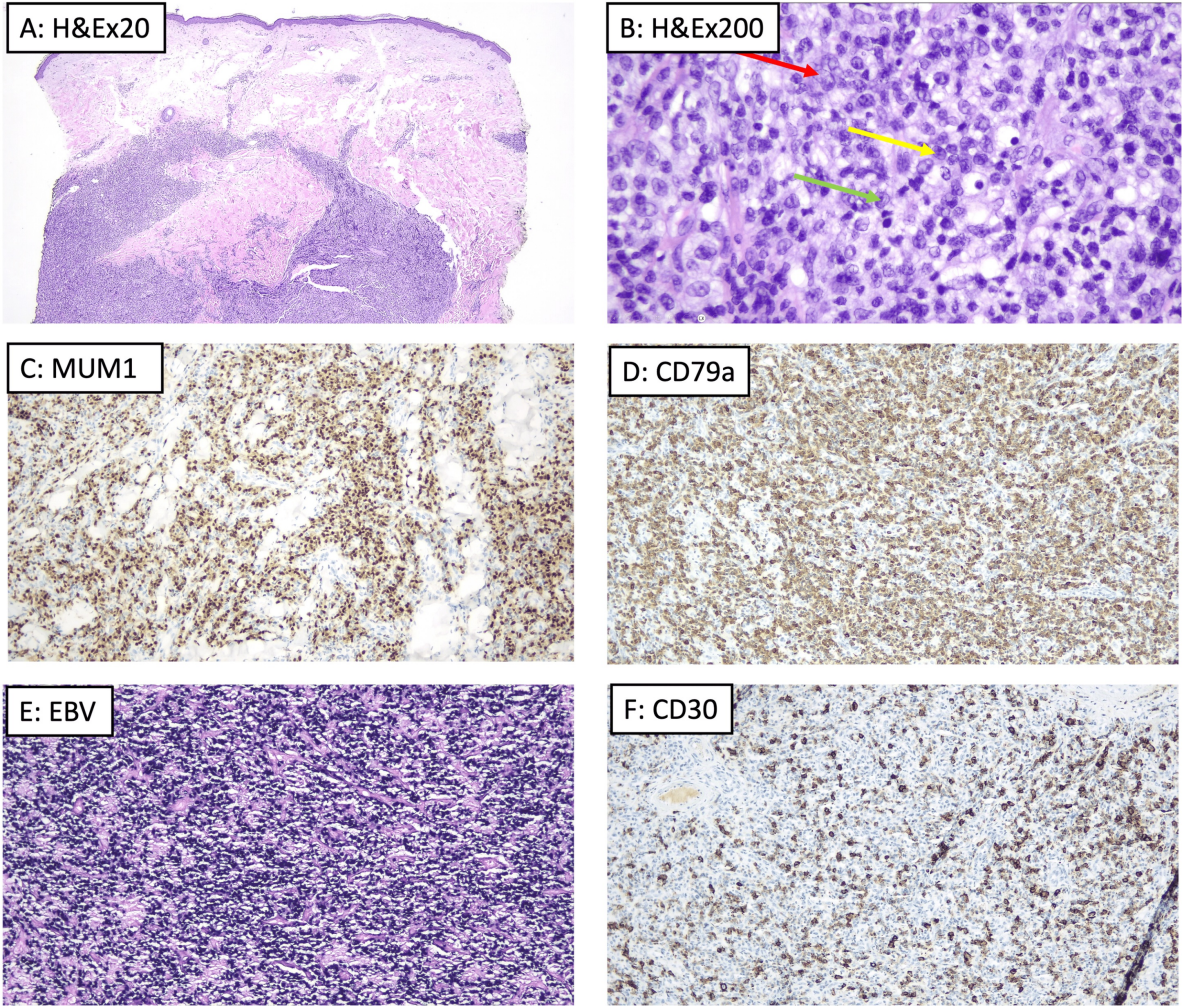
Histopathological and immunohistochemical features of a violaceous nodule suggestive of EBV-positive diffuse large B-cell lymphoma (DLBCL). (A) Low-magnification histology showing a dense non-epidermotropic infiltrate suggestive of B-cell lymphoma (hematoxylin and eosin {H&E}, ×20). (B) High-magnification histology revealing a predominance of large lymphocytes, including centroblasts (yellow arrow), immunoblasts (green arrow), and other atypical lymphocytes (red arrow) (hematoxylin and eosin, ×200). (C-F) Immunohistochemical staining demonstrating positivity for MUM1, CD79a, EBV (EBER by in situ hybridization), and CD30, characteristic of EBV-positive DLBCL. MUM1, multiple myeloma oncogene-1; EBV, Epstein-Barr virus; CD, cluster of differentiation; EBER, Epstein-Barr encoding region

A biopsy of a nodule on the lower limb showed a dense, non-epidermotropic infiltrate (Figure 2A) composed of large lymphocytes (Figure 2B, red arrow), including centroblasts (Figure 2B, yellow arrow) and immunoblasts (Figure 2B, green arrow). Immunohistochemistry (Figure 2C, 2D) demonstrated positivity for CD20 and CD79a, confirming B-cell lineage; additionally, there was positivity for multiple myeloma oncogene-1 (MUM1), B-cell lymphoma 6 (BCL6), and CD30, as well as for Epstein-Barr encoding region (EBER) by in situ hybridization. These findings were therefore consistent with EBV-positive DLBCL.

To evaluate systemic involvement, a full-body PET-CT scan was performed, revealing multiple adenopathies, including mediastinal, abdominal, pelvic, axillary, and inguinal. A biopsy of an inguinal lymph node identified a synchronous peripheral T-cell lymphoma, NOS. Bone marrow immunophenotyping confirmed marrow involvement by T-cell lymphoma. No evidence of systemic B-cell lymphoma was found, as both PET-CT and bone marrow immunophenotyping revealed no B-cell involvement beyond the skin. A summary of the immunophenotypic features supporting the distinction between the two lymphoma components is presented in Table 1.

**Table 1:** Immunophenotypic characterization of the two distinct lymphoma components. CD, cluster of differentiation; PD-1, programmed cell death protein 1; CXCR5, C-X-C chemokine receptor type 5; EBER, Epstein-Barr encoding region; MUM1, multiple myeloma oncogene-1

Site	Cell type/lymphoma subtype	Immunohistochemistry
Peripheral blood	Abnormal T-cell proliferation	CD4, CD5, CD3 (partial), CD7, CD2, CD28, CD27, CD45RO, CD25 (partial), PD-1 (CD279), and CXCR5
Inguinal lymph node and bone marrow biopsy	Small T-cell proliferation	CD3, CD5, CD2, CD7, and co-expression of CD4 and CD8 (slight CD4 predominance)
Skin biopsy	Large atypical B-cell proliferation	CD20, CD30, EBER (by in situ hybridization), CD79a, and MUM1

Based on these findings, we concluded that the patient had a primary cutaneous EBV-positive diffuse large B-cell lymphoma with a synchronous peripheral T-cell lymphoma, NOS.

**Figure 3:**
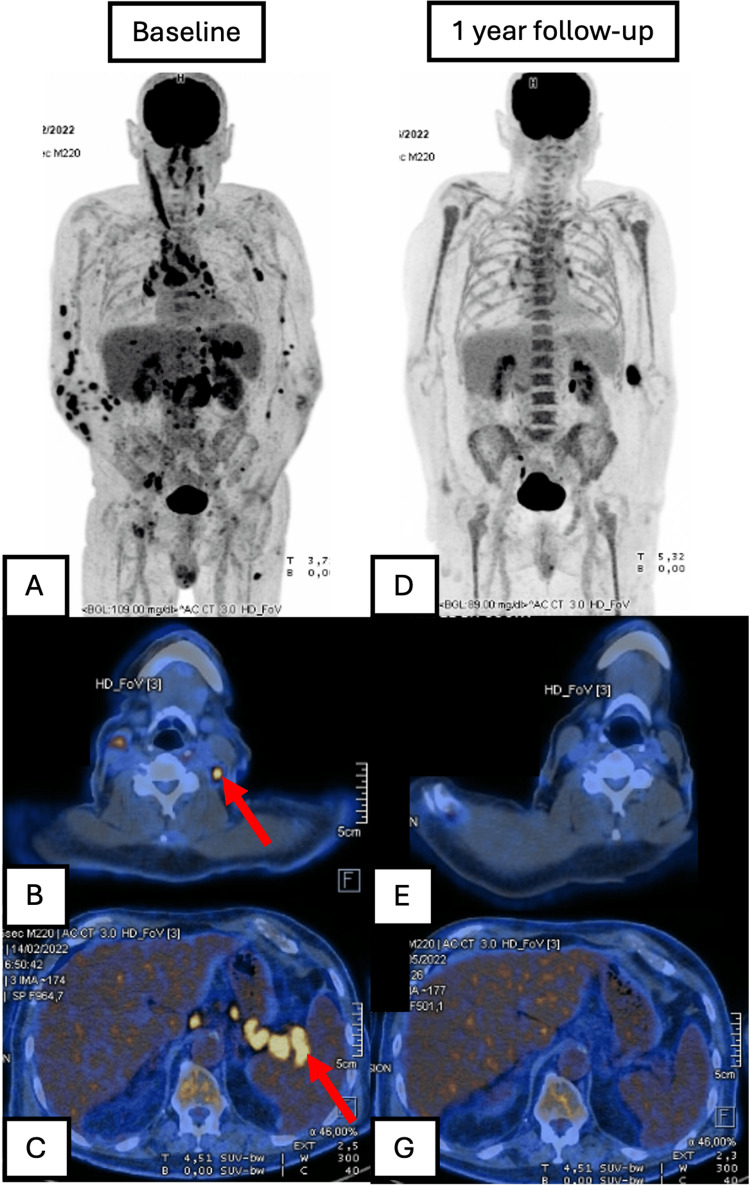
Baseline and 12‑month follow‑up FDG-PET-CT demonstrating complete metabolic response. (A) Whole‑body PET-CT scan at diagnosis reveals widespread hypermetabolic lymphadenopathy. (B) Axial PET-CT at the cervical level shows a left lateral cervical node (straight white arrow). (C) Coronal PET-CT below the diaphragm depicts multiple infra‑diaphragmatic adenopathies (straight white arrows). (D-F) Corresponding views obtained 12 months after six cycles of R‑CHOP: whole‑body (D), cervical (E), and infra‑diaphragmatic (F) images demonstrate complete metabolic response. FDG, fluorodeoxyglucose; R-CHOP, rituximab, cyclophosphamide, hydroxydaunorubicin, vincristine (Oncovin), and prednisone

**Figure 4:**
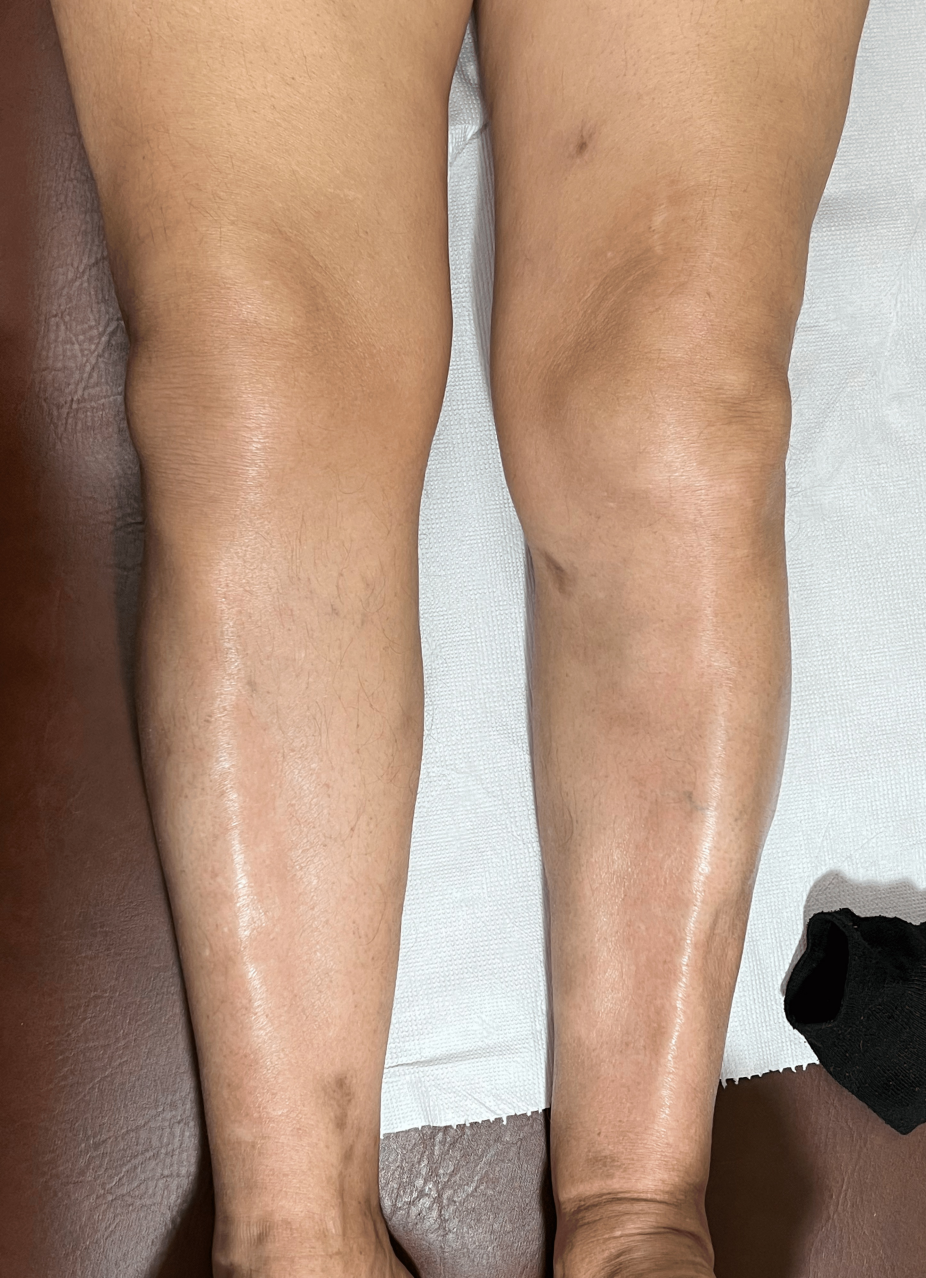
Follow-up, three months post treatment, with total resolution of the skin nodules.

The patient was referred to hematology and oncology and treated with R-CHOP chemotherapy (six cycles). End-of-treatment (EOT) fluorodeoxyglucose (FDG)-PET-CT revealed a complete metabolic response (Figure 3). This response was sustained after one year of follow-up (Figure 4).

## Discussion

This case underscores the importance of recognizing age as a significant risk factor for EBV-related malignancies [5-7]. The patient, aged 79, had no history of HIV infection, organ transplantation, or immunosuppressive therapy. However, besides age, the presence of a synchronous lymphoma, a rare association, may have further compromised his immune system. Despite the coexistence of a synchronous lymphoma, there was no systemic involvement of the B-cell lymphoma, supporting its classification as a primary cutaneous lymphoma.

In such cases, the detection of EBV through histological analysis and the presence of CD30 positivity are critical in differentiating EBV-positive DLBCL from other lymphomas, particularly diffuse large B-cell lymphoma, leg type [12]. Since these diagnostic tests are not routinely performed, EBV-positive DLBCL is likely underdiagnosed. The EBV-positive DLBCL is recognized as being more aggressive [8]. Although no standardized treatment guidelines currently exist, such distinctions could influence treatment decisions.

Data on the treatment of EBV-positive DLBCL remain scarce [12]. In this patient, R-CHOP chemotherapy was effective, achieving a complete metabolic response. Alternative treatments include interferon-alpha and radiotherapy [15]. Given the rarity of the association with a synchronous systemic T-cell lymphoma, evidence for optimal treatment strategies in such cases is lacking. This case supports the potential efficacy of R-CHOP for this rare dual lymphoma presentation.

Other reports of synchronous lymphomas also illustrate diagnostic and therapeutic complexity. For instance, Rana et al. (2025) described a case of synchronous DLBCL and classical Hodgkin lymphoma occurring at distinct anatomical sites, highlighting the diagnostic pitfalls when atypical morphology overlaps and molecular testing is limited [14]. Like our case, their diagnosis relied heavily on immunohistochemistry and histology from multiple biopsies. Treatment was initiated with a CHOP-like regimen, though the patient’s condition deteriorated rapidly. In contrast, early recognition and full-dose R-CHOP in our patient led to sustained remission. These rare cases reinforce the need for thorough investigation, site-specific biopsies, and broad immunophenotypic panels, including CD30 and EBER, to guide timely, effective therapy.

## Conclusions

Age should be considered a significant risk factor for EBV-related lymphomas. Diagnostic testing with EBER by in situ hybridization and CD30 should be considered in the evaluation of primary cutaneous diffuse large B-cell lymphomas to improve diagnostic accuracy and differentiation from other entities, namely, primary cutaneous diffuse large B-cell lymphoma, leg type. The awareness of possible synchronous lymphomas is essential, even in immunocompetent patients.

R-CHOP chemotherapy may represent an effective treatment option for the rare association of primary cutaneous EBV-positive DLBCL with synchronous systemic non-Hodgkin T-cell lymphoma, although further evidence is needed to establish optimal management strategies.
